# Cuts or carcasses? Diet form affects fecal microbial and animal fiber fractions in a large carnivore, the Asiatic lion

**DOI:** 10.1371/journal.pone.0335173

**Published:** 2025-10-22

**Authors:** Mengmeng Sun, Annelies De Cuyper, Yunhan Zhang, Marcus Clauss, Anouk Fens, Linda G. R. Bruins-van Sonsbeek, Geert P. J. Janssens

**Affiliations:** 1 Department of Veterinary and Biosciences, Ghent University, Belgium; 2 Clinic for Zoo Animals, Exotic Pets and Wildlife, University of Zurich, Switzerland; 3 Apenheul, the Netherlands; 4 Animal Nutrition Group, Department of Animal Sciences, Wageningen University and Research, the Netherlands; Tshwane University of Technology, SOUTH AFRICA

## Abstract

The care of exotic felids in zoos involves numerous factors, and the dietary management is currently considered particularly critical. This study investigated the effects of four different dietary regimens on the fecal microbiota and fecal characteristics of four female Asiatic lions (*Panthera leo persica*) at the Rotterdam Zoo, the Netherlands. The lions were sequentially fed beef meat on bone (BM01, 4 weeks), degutted and skinned cattle carcasses (CC, 4 weeks), degutted but unskinned banteng carcasses (BC, 2 weeks), and then returned to beef meat on bone (BM02, 4 weeks). Feces were collected at day 28, 56, 70 and 98, and represented the total feces of the group between the last feeding and the next feeding. 16S rRNA gene sequencing showed significant microbial shifts at phylum level, including, between CC and the subsequent diets, a decrease in Proteobacteria abundance and increases in Actinobacteria and Fusobacteria (p < 0.05). Fecal characteristics varied by diet. CC resulted in the highest proportion of visible bone, and BC in the lowest. Fecal particle size was highest on BC, and fecal volume on BC was about twice that of other feeding regimes, suggesting a dilution by indigestible skin and fur components. Significantly lower levels of fecal ash, calcium and phosphorus on BC (p < 0.001) supported the dilution hypothesis. On all diets, ash was shown to be a significant part of the animal fiber in the feces (p < 0.001). Although unskinned carcass may have required greater chewing effort, potentially increasing bone fragmentation, the reason for the reduced bone intake remains unclear. While the exact intake of animal fiber could not be quantified, differences in microbial composition and fecal characteristics were associated with variations in the type of animal fiber, such as bone versus skin.

## Introduction

The management of exotic felids in zoos involves a multitude of factors, with nutritional management standing out as paramount [1]. A survey of the feeding practice of large carnivores in 44 European zoos found that meat on bone and small animal carcasses (such as rabbits or chickens) are the main dietary items, but large animal carcasses are rarely fed [2]. Several studies have demonstrated the effects of carcass feeding on the physical and mental health of zoo carnivores. For instance, raw meat diets fail to allow the feeding behavior of wild carnivores when consuming prey [3]. Oral health assessments of wild and zoo lions and tigers have shown significantly higher rates and severity of dental calculus and periodontal disease in individuals when under human care. This is attributed to the abrasive properties of carcass diets in the wild, where bones, tendons, and skin contact the teeth, reducing the incidence of oral diseases [4]. The lack of hunting-related stimulation under human care often leads to stereotypic behaviors such as excessive grooming, lethargy, and pacing [5]. A long-term study of a female Asiatic lion (*Panthera leo persica*) at Chester Zoo reported a significant reduction in pacing behavior and increased feeding and resting times after a 12 month carcass feeding program [6], and a similar finding was reported for two tigers during an experiment that included the provision of a large carcass followed repeatedly by 10 day fasting periods [7]. Additionally, and logically, two studies showed that the actual time spent feeding is longer for whole carcasses than for pieces of meat [8,9].

Whole carcass feeding includes a broader range of animal tissues such as bones, tendons, cartilage, skin, hair, and feathers – termed “animal fiber” [10]. The presence of the latter serves as a modulator of gut fermentation and plays a role in modulating fecal microbiota composition. Cheetahs fed a whole rabbit diet exhibited lower levels of putrefaction and inflammation compared to those fed a meat supplemented diet, characterized by significantly reduced concentrations of propionate, butyrate, calprotectin and S100A12 in feces, as well as indoxyl sulfate in serum [10,11]. More *Clostridiaceae* were detected in the feces of carnivores on a natural diet based on animal carcasses (cheetah [12] and domestics cat [13]). However, few studies have compared the effects of different form of raw food with different characteristics on the gut microbiota, ranging from meat on bone cuts to whole carcasses, potentially resulting in variation in the actual intake of fibrous particles. The effect of ingesta particle size in digestive physiology has mainly been investigated in herbivores. One way of studying herbivore chewing efficacy is analyzing feces for their mean particle size [14], because in large terrestrial herbivores, digestive processes have comparatively little influence on changes in digesta particle size during passage through the gastrointestinal tract. The whole carcass feeding paradigm provides another perspective to study the effects of particle size in carnivores. This diet contains a variety of components such as skin, hair, and bone, which significantly increases the complexity of dietary particles.

In the present exploratory study, we took the opportunity to follow a set of diet changes in a lion group at Rotterdam Zoo, the Netherlands, to compare different forms of raw diets in zoo lions and explore their potential effects on fecal microbiota and fecal characteristics, including fecal animal fiber content and fecal particle size.

## Materials and methods

### Ethics statement

The research focused on non-invasive sample collection, specifically fecal samples, from Asiatic lions at Rotterdam Zoo, the Netherlands. No invasive procedures or interventions were performed on the animals during the study. As the research did not involve any invasive techniques, no additional ethical approval was required. All animals were cared for in strict compliance with relevant animal welfare regulations, including the European Convention for the Protection of Animals Used for Scientific Purposes (Directive 2010/63/EU).

### Animals and diets

Four female Asiatic lions (*Panthera leo persica*) at the Rotterdam Zoo in the Netherlands participated in the study (03.05.- 07.08.2022), i.e., the mother (birth date 18.04.2010) and her three adult daughters (birth date 21.08.2018). The animals shared an indoor and outdoor space. No apparent medical or health issues were reported. In the first period, the regular diet was given: beef meat on bone (BM01), with an average of 10 kg per animal fed twice a week for 4 weeks, i.e., a total of 80 kg per week. Food pieces were scattered throughout the enclosure so that the animals could eat individually. The second period provided half a cattle (*Bos bovis*) carcass (CC) without skin, hair or internal organs, averaging 113 kg (range 105–125 kg) fed once a week for 4 weeks. In the third period, half a banteng (*Bos javanicus*) carcass (BC) was given, containing skin and hair but no internal organs, averaging 98 kg (range 95–100 kg) fed once a week for 2 weeks. All carcasses were offered in one piece and lions had to share food. In the fourth and final period, all lions were fed again the beef meat on bone (BM02), with an average of 10 kg per animal fed twice a week for 4 weeks.

### Sample collection

The total weight of food was recorded before feeding each week, and leftover food was collected in the enclosure one week later and weighed to estimate weekly food intake. No correction for moisture loss was applied. Analyzing representative samples of food, and of the leftovers, was beyond the logistical means of this study. Before feeding, enclosures were cleaned of feces and leftover food, after which lions had unrestricted access for a week without additional feeding or cleaning.

Feces were collected at day 28 (BM01), 56 (CC), 70 (BC) and 98 (BM02), representing the total fecal output from all four lions between the last feeding and the next feeding (i.e., from the preceding week). Hence, the feces varied in freshness with a maximum of one week in the environment before collection. A total of 34 feces samples were collected for analysis, with seven, six, thirteen, and eight feces samples collected at the four time points, respectively. The fecal samples were weighed and scored on a five-point visible scale where one is classified as ‘bullet like’ and five as ‘entire liquid stool’ [15]. Approximately 1 g of subsample was added to pre-weighed 2 ml safety lock tubes (Eppendorf AG, Germany) and stored at −80°C until DNA extraction. The remaining feces were pre-processed to separate the undigested animal tissues including skin/hair and bones visible in the feces, weighed separately to calculate their percentage in the total feces, and stored at −20°C for subsequent analysis. Feces with visible skin/hair and bones removed are referred to as ‘sorted feces’ in the subsequent analyses. The concentrations of dry matter (DM), animal fiber, ash and minerals for a whole fecal sample calculated by adding the contents of the three fecal components (sorted feces, bone, and skin), weighted according to their proportion in the total feces.

### Microbiota analysis

DNA extraction was conducted with the Qiagen DNeasy® PowerLyzer® PowerSoil® Kit (ref 12855−100), and the concentration was measured using the Nanodrop ND-1000 spectrophotometer (Nanodrop Technologie). The extracted DNA samples were stored at −80°C until further analyses. Ten µl genomic DNA extract was sent to LGC genomics GmbH (Germany), where the 16S rRNA gene V3-V4 hypervariable region was amplified following the protocol described by Van Landuyt et al (2020). The PCR mix consisted of 1 µl of 10x diluted DNA extract, 15 pmol of both the forward primer 341F 5’- NNNNNNNNNTCCTACGGGNGGCWGCAG and reverse primer 785R 5’- NNNNNNNNNNTGACTACHVGGGTATCTAAKCC [16], in a 20 µl volume of MyTaq buffer containing 1.5 units MyTaq DNA polymerase (Bioline) and 2 µl of BioStab II PCR Enhancer (Sigma-Aldrich, USA). Each sample was tagged with a unique 10-nt barcode sequence on both the forward and reverse primers. PCRs were carried out for 30 cycles under the following conditions: 2 min at 96°C for pre-denaturation; followed by 96°C for 15 s, 50°C for 30 s, and 70°C for 90 s.

DNA bands of amplicons of interest were determined by gel electrophoresis. Approximately 20 ng of amplicon DNA concentration from each sample was pooled with up to 34 samples carrying different barcodes. The amplicon pools were purified with one volume of AMPure XP beads (Agencourt) to remove primer dimer and other small mispriming products, followed by an additional purification step using MinElute columns (Qiagen). Finally, approximate 100 ng of each purified amplicon pool DNA was used to construct Illumina libraries through adaptor ligation using the Ovation Rapid DR Multiplex System 1–96 (NuGEN). The Illumina libraries were pooled and size selected using preparative gel electrophoresis, and sequencing was performed on an Illumina MiSeq using v3 Chemistry (Illumina, USA) with a read length of 2 × 300 bp.

### Bioinformatics data processing

The amplicon sequence data were processed using the DADA2 R package following the pipeline tutorial [17]. In the quality control step, primer sequences were removed, and reads were truncated based on quality scores (truncQ = 2). Additional filtering was performed to remove reads containing ambiguous bases or those exceeding the maximum expected error threshold (maxEE = 2.2). After dereplication, unique sequences were denoised using the DADA with the selfConsist sample inference method (pooling = TRUE). The error rates were estimated and visually inspected before proceeding with paired-end read merging, ensuring a minimum overlap of 20 bp and no mismatches (maxMismatch = 0). Chimeric sequences were removed using the consensus method, and the final amplicon sequence variant (ASV) table was generated. Taxonomy was assigned using the Naïve Bayesian Classifier and the DADA2-formatted Silva v138 database [18]. To exclude non-microbial sequences, ASVs classified as chloroplasts or mitochondria were removed prior to downstream analyses. Additionally, singletons (ASVs with a total abundance of 1 across all samples) were filtered out to reduce potential sequencing artifacts.

### Animal fiber analysis

The sorted feces, pre-selected visible bone, and skin/hair were freeze-dried using a CoolSafe™ lyophilizer (SCANVAC, Denmark) to determine DM separately. To quantify the animal fiber content of these materials, we followed the amylase-treated neutral detergent residue including animal fiber (aNDRa) method described by D’Hooghe *et al.* [19]. Forty-four freeze-dried samples included sorted feces (n = 34), visible bones (n = 8, two random samples were selected from each of the four diet groups), and visible skin/hair (n = 2) from the BC diet. All samples were ground with a 2.0 mm screen in a centrifugal mill (Retsch ZM 200, 12,000 rpm). F57 filter bags (ANKOM technology, USA) were pre-weighed (W1: weight of empty filter bag) and filled with approximately 0.5 g of sample (W2: sample weight) in duplicate, then sealed using a heat sealer. Fat was removed by soaking the bags in acetone for 2 x 10 mins, followed by overnight drying in a fume hood. The filter bags were placed in an ANKOM200 Fiber Analyzer (ANKOM Technology, USA). Protease (5.0 mg per 0.5 g of sample) was added to 2 l phosphate buffer, and the samples were refluxed for 90 mins at 40°C. After rinsing with distilled water, a neutral detergent solution was added along with alpha-amylase. The samples were refluxed for 75 mins at 100°C, rinsed twice with boiling water and alpha-amylase, and once more with boiling water. The filter bags were dried overnight under a fume hood, then placed in an oven at 103°C for 4 hours. After cooling in a desiccator, the samples were weighed (W3: dry weight of bag with fiber after extraction). Blanks were also measured for correction: C1 is the average weight of two empty bags, and C2 is the average weight of the final oven-dried empty bags. The amylase-treated neutral detergent residue including animal fiber fraction without ash correction (aNDRa; in % DM) was calculated by the following formula:


$$\text{a}NDR\text{a}=\frac{\left(W\text{3}-\left(W\text{1}\times C\text{2}/C\text{1}\right)\right)\times \text{1}\text{0}\text{0}}{W\text{2}}$$

### Ash analysis

The total ash (TA) content of the 44 samples pre-treated by freeze-drying and grinding mentioned above was determined. Approximately 2 g of sample were placed in a pre-incinerated crucible of known weight. The crucible was pre-incinerated at 450°C for 30 mins and then ashed in a muffle furnace at 600°C for 4 hours. The total ash content was expressed as % DM. The TA was expressed as % of dry matter (DM) and calculated as:


$$TA=\frac{W_{crucible+ash}-W_{crucible}}{W_{feces}}\times \text{1}\text{0}\text{0}$$

where *W*_*crucible+ash*_ is the weight of the crucible with ash after ashing, *W*_*crucible*_ is the weight of the crucible before use, and *W*_*feces*_ is the weight of the feces sample before ashing.

The neutral detergent insoluble ash (NDIA) was determined using the same principle after animal fiber analysis and expressed as % of DM:


$$NDIA=\frac{W_{crucible+NDIA}-W_{crucible}}{W_{NDF\;residue}}\times AF_{\%DM}$$

where W_crucible+NDI_ is the weight of the crucible with NDIA after ashing, W_crucible_ is the weight of the crucible before use, W_NDF residue_ is the weight of the neutral detergent residues after amylase treatment in the animal fiber fraction, and AF_%DM_ is the animal fiber content expressed as % of DM.

The acid insoluble ash (AIA) analysis in feces was performed according to the method of Liu [20]. Empty 50 ml plastic centrifuge tubes (nerbe plus GmbH & Co. KG, Germany) were placed in a forced-air oven at 95°C for 30 mins, cooled to room temperature, and weighed. The TA residue resulting from the TA analysis described above was ground using a mortar and pestle, mixed thoroughly, and 100 mg was weighed into pre-weighed centrifuge tubes. Twenty ml of 2N HCl was added to each tube, the lids were secured, and the tubes were incubated in a shaking incubator (KS 4000 i control, Germany) at 80°C and 250 rpm for 30 mins. After incubation, the tubes were cooled for 10 mins, centrifuged at 3700 × g for 10 mins, and the supernatant was carefully discarded. The pellet was washed twice by adding 20 ml of deionized water, vertexing, and centrifuging. The uncapped centrifuge tubes containing the pellet were placed in a forced-air oven at 95°C for 2 hours to dry. Finally, the tubes were cooled to room temperature and weighed. The AIA content measured was first calculated and expressed as % of TA and then converted to % DM in feces by multiplying the value with the fecal TA content (in % DM). The calculation was as follows:


$$AIA=\frac{W_{tube+AIA}-W_{tube}}{W_{ash}}\times TA_{\%DM}$$

where W_tube+AIA_ is the weight of the sample tube with AIA after centrifugation and drying, W_tube_ is the weight of the sample tube before use, W_ash_ is the weight of ash used for mixing with 2 N HCl,

and TA_%DM_ is the fecal TA content expressed as % of DM.

### Mineral analysis

Mineral content, including calcium (Ca) and phosphorus (P), was quantified using inductively coupled plasma optical emission spectrometry (ICP-OES, Thermo Scientific iCAP 7000 Duo). Filters containing 1g of same 44 samples were placed in pre-tared 50 ml digestion tubes, then digested with 6 ml of nitric acid (69%) and 1.5 ml of hydrochloric acid (37%) at 105°C for 2 hours. After cooling, the samples were diluted to 50 ml with ultrapure water and filtered through a 0.45 μm syringe filter to remove particulates. Elemental analysis was conducted in two batches: one for Ca, the other for P. Calibration standards ranging from 0 to 50 mg/l were prepared using single-element stock solutions, and a 2 mg/l quality control standard, with yttrium (Y) at 2 mg/l as an internal standard, was used to monitor instrument stability. Multiple wavelengths were analyzed in both radial and axial viewing modes for each element, and results were cross-validated to eliminate interferences and ensure data accuracy.

### Fecal particle size analysis

Feces without visible skin/hair and bones were mixed and analyzed for fecal particle size by wet sieving as described by Fritz *et al.* [21]. A Retsch AS 200 digit sieve machine (Retsch, Haan, Germany) with sieve sizes 5, 1, 0.5, 0.3, and 0.02 mm (linear dimensions of holes) was used at a water throughput of 2l/min; sieving time was 8 mins at a vibration intensity of 2 mm. Particles passing the finest sieve were discarded. The particles of each fraction were transferred onto pre-weighed petri dishes, dried at 60°C for 24 hours, and weighed after cooling to room temperature in an exsiccator. Particles smaller than 0.02 mm are calculated as the total dry weight of the sieved sample minus the weight retained on each cascade sieve after drying. The undigested skin/hair and bones that were removed during pre-processing in selected feces and freeze-dried (see above) were directly sieved using sieves with diameters of 100, 50, and 25 mm, and the weight of the sample retained on each sieve was weighed separately.

The approaches presented here are based on mass measurements and hence on average particle size per unit mass, not per number of particles. The mean particle size (MPS) is calculated according to the method of Fritz *et al*. [21]. The MPS is defined as the weighted average (here called the discrete average dMEAN) of the sieves sorted from size *S*(1) (minimum) to size *S(n)* (maximum pore size). The fraction *p(i)* of particles retained at size *S(i)* includes particles smaller than *S(i + 1)* but excludes particles smaller than *S(i)* (“cumulative oversize”). The obvious estimate of the mean size of these particles is the average between *S(i + 1)* and *S(i)*. If the maximum particle size *S(n + 1)* is estimated in advance, the mean size of the particles retained at the largest sieve aperture size *S(n)* can be estimated in the same way. dMEAN can then be obtained by multiplying these mean sizes by the corresponding fractions and adding the results:


$$dMEAN=\sum_{i=\text{1}}^{n}p(i)\times \frac{S(i+\text{1})+S(i)}{\text{2}}$$

### Statistical analysis

Microbial taxonomic composition and differential abundance analyses were conducted in RStudio v4.1.2 [22], using packages ggplot 2 3.3.5 [23] for graph visualization. Alpha diversity indices (Richness (observed ASV), Shannon, Invsimpson) were calculated on ASV after normalization by scaling with ranked subsampling (SRS) [24], using the phyloseq v1.42.0 [25] package. Beta diversity was assessed with phyloseq and vegan v2.6-4 [26] based on Bray–Curtis dissimilarity, and visualized using principal coordinate analysis (PCoA) plots. Group differences were tested using permutational multivariate analysis of variance (PERMANOVA), with significance determined at p < 0.05. The normality of residuals was evaluated using residual vs. fitted plots and Q–Q plots. To evaluate within-group variability, descriptive variance analysis was performed at both phylum and genus levels by calculating mean values, standard deviations (SD), and variances across samples belonging to the same dietary group. These calculations were conducted using R software (v4.3.2). Since some microbial composition variables did not follow a normal distribution, non-parametric methods were applied. At the phylum level, the top 10 most abundant taxa were compared across the four dietary groups using the Kruskal–Wallis H test. For phyla with significant differences (p < 0.05), pairwise comparisons were further conducted using the Mann–Whitney U test. The same approach was applied to the top 20 genera. False discovery rate correction was applied using the Benjamini–Hochberg method (FDR-BH), with adjusted significance set at p _adj_ < 0.05. These analyses and visualization were conducted using GraphPad Prism 10.0.

For fecal characteristics, residuals were assessed and found to conform to a normal distribution. One-way analysis of variance (ANOVA) was used to compare means across dietary groups, followed by Tukey’s post hoc test for multiple comparisons, with significance set at p < 0.05. To compare the differences in animal fiber measurements between the same fecal sample with and without ash correction and to evaluate the effect of diet, repeated measures were conducted using SPSS 29.0. The dependent variable was animal fiber, with ash condition (with or without ash) as the within-subject factor and diet type as the fixed factor. The model also included the ash × diet interaction. Individual samples were treated as a random effect to account for the repeated measurements. Mauchly’s test of sphericity was used to assess the sphericity assumption. If violated, the Greenhouse-Geisser correction was applied. A significance level of α = 0.05 was used. For significant main effects or interactions, post-hoc tests were conducted to further compare the differences between groups. All fecal parameter results were visualized using GraphPad Prism 10.0.

Spearman’s correlation analysis was performed in IBM SPSS 29.0 to assess relationships between fecal characteristics and microbial taxa. The strength and direction of associations are indicated by Spearman’s rank correlation coefficient (*r*), with positive and negative correlations visualized in blue and red, respectively. Statistical significance was set at p < 0.05.

## Results

### Fecal bacteria composition and relative abundance

**Fig 1:**
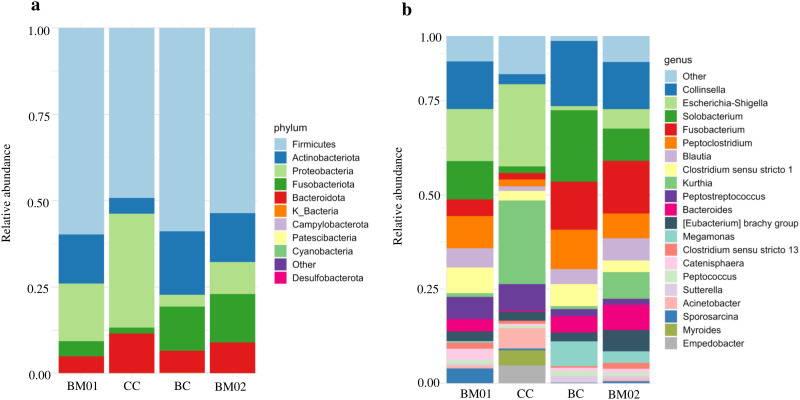
Fecal microbiota analysis of zoo-housed Asiatic lions fed beef meat on bone (BM01 and 02), cattle carcass (CC), and banteng carcass (BC). Values represent mean relative abundances per dietary group. **a**. The top 10 most abundance phyla were selected, and the remaining ones were classified as “Other”; **b**. The top 20 most abundance genera were selected, and the remaining ones were classified as “Other”.

A total of 1,068,673 amplicon sequences based on the 16S rRNA gene were obtained, with an average of 30,534 ± 13,748 reads per fecal sample (range = 4,170–64,141). Fig 1 illustrates the relative abundance of the microbial communities at the phylum and genus levels. At the phylum level, Firmicutes was the most dominant across all four diets (59.8% ± 14.6%, 49.3% ± 31.0%, 54.6% ± 20.6%, and 53.7% ± 14.0%, respectively). In the BM01 and CC diets, Proteobacteria was the second most abundant phylum, followed by Actinobacteria and Bacteroidetes. For the BC and BM02 diets, Actinobacteria and Fusobacteria were the second and third most abundant phyla, respectively. At the genus level, there was variation in fecal microbiota composition across the four diets. The first most abundant genus for each diet was *Escherichia-Shigella* (13.8% ± 23.1%), *Kurthia* (22.2% ± 21.8%), *Solobacterium* (19.0% ± 15.7%), and *Fusobacterium* (14.0% ± 9.9%), respectively. The second most abundant genus across all diets was *Collinsella* (except for the CC diet, where *Escherichia-Shigella* was second). The third most abundant genera were *Solobacterium* in BM01 and BM02, *Peptostreptococcus* in CC, and *Fusobacterium* in BC. Variance analysis further indicated that within-group variability was generally moderate compared to between-group differences (S1 Table). To reflect individual level variation, relative abundances of bacterial phyla and genera for each fecal sample are provided in S1 and S2 Figs, respectively.

### Diversity of fecal microbiota

**Fig 2:**
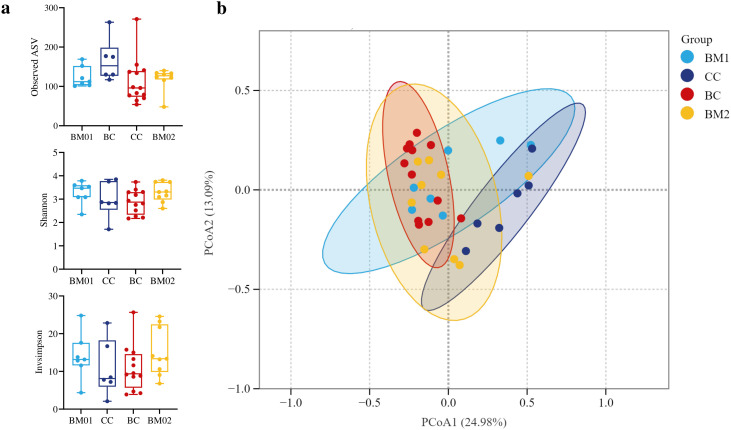
Diversity of fecal microbiota of zoo-housed Asiatic lions fed beef meat on bone (BM01 and 02), cattle carcass (CC), and banteng carcass (BC). **a**. Alpha diversity measures. Shannon and Invsimpson index with median and interquartile ranges. **b**. Principal coordinate analysis (PCoA) plot based on Bray–Curtis dissimilarities of microbial community structure (PERMANOVA, R^2^ = 0.205, p _adj_ < 0.001).

Fig 2 shows the alpha diversity and beta diversity during different periods. Observed ASV, Shannon and Invsimpson index comparison showed that different diets did not significantly affect alpha diversity at ASV levels. Beta diversity, as measured by Bray–Curtis dissimilarities, showed microbial communities differed significantly across the four dietary treatments (PERMANOVA, R² = 0.205, p _adj_ < 0.001). Although there was partial overlap among the groups, CC and BC showed a separation.

### Fecal microbiota dynamics in lions switching diet

**Fig 3:**
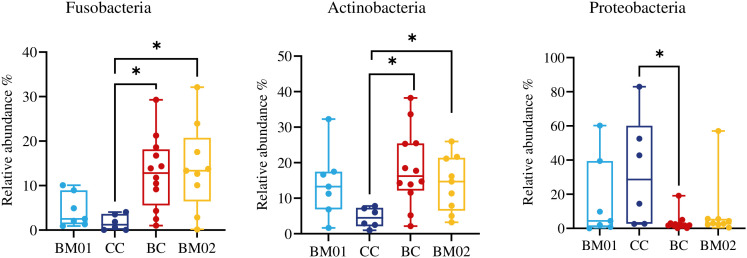
Changes in relative abundance of fecal microbiota at phylum level in feces of zoo-housed Asiatic lions fed beef meat on bone (BM01 and 02), cattle carcass (CC), and banteng carcass (BC). Multiple testing was performed using the Mann Whitney U tests with Benjamini–Hochberg false discovery rate correction (an asterisk means that p _adj_ < 0.05).

During the lions’ dietary transition, significant differences in microbial composition were observed at both the phylum (Fig 3) and genus levels (S3 Fig). At the phylum level, switching the diet from BC to CC led to a significant decrease in Proteobacteria (from 33.0% ± 32.1% to 4.4% ± 6.1%, p _adj_ = 0.0282), while Actinobacteria and Fusobacteria significantly increased (from 4.6% ± 2.8% to 17.0% ± 11.4%, and 1.7% ± 1.8% to 11.8% ± 8.6%, respectively; p _adj_ = 0.0360 and 0.0120). When the diet was switched back to beef meat (BM02), both Fusobacteria and Actinobacteria remained significantly more abundant than in the CC group (p _adj_ = 0.0360).

At the genus level, 9 out of the 20 most abundant genera exhibited significant pairwise differences across dietary groups. For instance, *Acinetobacter* and *Kurthia* were significantly enriched in the CC group compared to BM01, BC, or BM02. In contrast, *Collinsella*, *Fusobacterium*, *Megamonas*, *Peptoclostridium*, and *Solobacterium* were more abundant in the BC or BM02 groups. *Bacteroides* increased in BM02 compared to BM01, while *Peptostreptococcus* decreased in BC relative to BM01. Exact adjusted p values are shown in the corresponding figure (S3 Fig).

### Food intake and fecal characteristics

**Fig 4:**
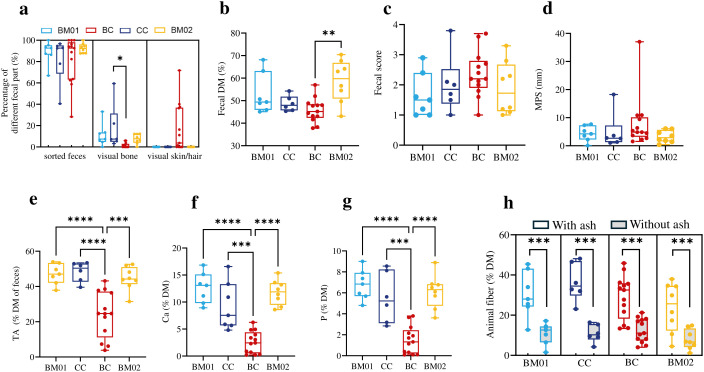
Changes in fecal characteristics of zoo-housed Asiatic lions fed beef meat on bone (BM01 and 02), cattle carcass (CC), and banteng carcass (BC). **a**. The percentage of sorted feces, visible bone, and visible skin/hair. **b**. The fecal dry matter (DM). **c**. The fecal score. Scored on a five-point visible scale where 1 is classified as ‘bullet like’ and 5 as ‘entire liquid stool’. **d**. The mean particle size (MPS). **e**. Fecal total ash (TA). **f.** The fecal calcium (Ca). **g.** The fecal phosphorus **(P)**. **h.** Fecal animal fiber (with or without ash). Statistically significant differences among the four dietary groups were assessed using the one-way analysis of variance (ANOVA) test (an asterisk means that p < 0.05; two asterisks means that p < 0.01; triple asterisks means p < 0.001; four asterisks means p < 0.0001).

The proportion of visual bone in feces (Fig 4a), with BC feces having less visual bones (p = 0.0251). Fecal DM content (Fig 4b) significantly increased from the BC to BM02 (p = 0.0034). Fecal score and MPS were not significantly affected by the dietary type, even though BC had the highest numerical values (Fig 4c, 4d). BC feces were significantly lower in TA (Fig 4e), Ca (Fig 4f) and P (Fig 4g) than feces on other diets. BC feces had a significantly higher proportion of AIA in total ash than the feces on the other diets (S2 Table). There were no differences between diets in fecal animal fiber content (Fig 4h). For the animal fiber content, a significant effect of whether ash content was controlled for or not was observed (p < 0.001). However, the interaction between ash × diet type was not significant (p = 0.0639), indicating a similar effect of ash correction across diets. The NDIA generally represented more than half of the uncorrected animal fiber value (Fig 4h).

The BC diet resulted in higher fecal output, with more than twice the total number of fecal deposits per week than CC, alongside a marked rise in visible skin and hair residues (252 g/week). Correspondingly, the total weekly excretion of fecal animal fiber was also distinctively higher on BC than on the other diets (Table 1). The weekly fecal TA excretion was similar among the four diets, but the total amount of visible bones as well as fecal Ca and P excretion were lowest in the BC diet, indicating that on BC, less bone was ingested. The weekly fecal AIA excretion was higher on the carcass diets (CC and BC) than on the cut meat diets (BM01 and BM02), suggesting a higher intake of soil or dust on the carcass diets, with the highest effect for the skin-covered carcass BC.

**Table 1:** Characteristics of diets, fecal samples collected from zoo-housed Asiatic lions fed beef meat on bone (BM01 and BM02), cattle carcass (CC), and banteng carcass (BC), and weekly total excretion of various substances via feces.

Collection time	D 28	D 56	D 70	D 98
Food type	BM01	CC	BC	BM02
Estimated average food intake (kg fresh matter/week)	68	102	89	68
Total number of feces (/week)	7	6	13	8
Total feces, fresh matter (g/week)	2222	2265	4419	1942
Defecation weight ^a^, fresh matter (g)	317 ± 108	377 ± 151	340 ± 218	243 ± 112
***Weekly total DM weight (g)***				
Defecation weight ^a^	165 ± 57	185 ± 80	160 ± 110	137 ± 49
Whole feces	1154	1113	2083	1093
Sorted feces ^b^	1043	997	1777	1009
Visible bone	111	116	54	84
Visible skin/hair	0	0	252	0
Animal fiber with ash	344	379	599	259
Animal fiber without ash	127	107	257	86
Ca + P	214	167	107	191
TA	531	529	544	481
NDIA	218	274	340	174
AIA	79	166	253	93

DM, dry matter; Ca, Calcium; P, Phosphorous; TA, total ash; NDIA, neutral detergent insoluble ash; AIA, acid insoluble ash; SD, standard deviation.

^a^Mean ± SD

^b^Feces with visible skin/hair and bones removed are referred to as ‘sorted feces’

### Correlations between fecal characteristics including microbiota

Fecal DM was negative correlated to the percentage of visible skin (Table 2), suggesting that a higher percentage of skin led to increased fecal moisture (r = −0.50; p = 0.0074). The percentage of visible skin in feces was positively correlated with fecal MPS (r = 0.47; p = 0.0048), but no systematic correlation was found between fecal score and MPS. The percentage of visible skin in feces and the fecal score generally showed a negative, and fecal DM a positive relationship with indicators of bone (% visible bone, TA, Ca + P) (Table 2).

**Table 2:** Correlation matrix of fecal characteristics (concentrations) and Microbial Taxa (relative values) in zoo-housed Asiatic lions (*Panthera leo persica*).

	Fecal DM	Fecal score	MPS	Visible Bone	Visible skin	TA	NDIA	AIA	TA-NDIA	TA-AIA	Ca + P	Animal fiber with ash	Animal fiber without ash	Actinobacteria	Bacteroidetes	Firmicutes	Fusobacteria	Proteobacteria
Fecal DM	1.00																	
Fecal score	−0.29	1.00																
MPS	−0.40^*^	0.03	1.00															
Visible Bone	0.37^*^	−0.25	0.11	1.00														
Visible skin	−0.45^**^	0.23	0.47^**^	−0.50^**^	1.00													
TA	0.47^**^	−0.36^*^	−0.51^**^	0.50^**^	−0.72^***^	1.00												
NDIA	0.22	0.09	−0.12	0.44^**^	−0.45^**^	0.44^**^	1.00											
AIA	0.03	0.42^*^	−0.14	−0.04	−0.19	−0.10	0.49^**^	1.00										
TA-NDIA	0.52^**^	−0.44^**^	−0.48^**^	0.48^**^	−0.69^***^	0.82^***^	−0.03	−0.28	1.00									
TA-AIA	0.52^**^	−0.48^**^	−0.46^**^	0.56^***^	−0.71^***^	0.92^***^	0.22	−0.36^*^	0.90^***^	1.00								
Ca + P	0.52^**^	−0.51^**^	−0.43^*^	0.58^***^	−0.70^***^	0.91^***^	0.20	−0.34^*^	0.91^***^	0.98^***^	1.00							
Animal fiber with ash	0.10	0.12	0.14	0.32	−0.18	0.19	0.89^***^	0.36^*^	−0.26	0.02	−0.03	1.00						
Animal fiber without ash	−0.11	0.13	0.46^**^	0.14	0.17	−0.21	0.53^**^	0.11	−0.49^**^	−0.27	−0.30	0.83^***^	1.00					
Actinobacteria	0.28	0.04	−0.02	−0.15	0.10	−0.06	−0.12	0.13	0.08	−0.04	−0.05	−0.06	−0.11	1.00				
Bacteroidetes	−0.18	0.21	0.06	−0.22	0.09	−0.12	0.01	0.04	−0.19	−0.17	−0.20	0.06	0.12	−0.15	1.00			
Firmicutes	0.05	0.09	0.04	0.10	−0.05	−0.20	−0.03	0.12	−0.06	−0.14	−0.13	−0.05	−0.08	0.13	−0.35^*^	1.00		
Fusobacteria	0.03	0.25	0.17	−0.27	0.18	−0.37^*^	−0.29	−0.03	−0.22	−0.25	−0.28	−0.25	−0.20	0.24	0.20	0.12	1.00	
Proteobacteria	0.10	−0.13	−0.29	0.16	−0.23	0.41^*^	0.19	−0.10	0.25	0.36^*^	0.35^*^	0.09	0.01	−0.57^***^	0.17	−0.61^***^	−0.31	1.00

DM, dry matter (% fresh matter); MPS, mean particle size (mm); TA, total ash (% DM); NDIA, neutral detergent insoluble ash (% DM); AIA, acid insoluble ash (% DM); Ca, calcium (% DM); P, phosphorus (% DM). The analysis was conducted using Spearman’s rank correlation coefficient. Blue indicates positive correlations, while red indicates negative correlations. The intensity of the color represents the strength of the correlation, with r values shown in the table. Statistically significant correlations are marked as follows: p < 0.05 (*), p < 0.01 (**), p < 0.001(***).

The percentage of visible bone was positively correlated with fecal Ca and P (r = 0.58; p < 0.001) but negatively correlated with the percentage of visible skin (r = −0.50; p = 0.0024). A significant negative correlation was observed between visible skin and the sum of fecal Ca and P (r = −0.70; p < 0.001). Visible skin also showed significant negative correlations with ash-related parameters, including TA (r = −0.72; p < 0.001), NDIA (r = −0.45; p = 0.0080), TA minus NDIA (r = −0.69; p < 0.001), and TA minus AIA (r = −0.71; p < 0.001). The ash in animal fiber, NDIA, correlated positively with both AIA (r = 0.49; p = 0.0034) and TA (r = 0.44; p = 0.0092). Total ash minus NDIA correlated positively with TA minus AIA (r = 0.90; p < 0.001). Across diets, there was a positive correlation between the sum of fecal Ca and P and TA (r = 0.91; p < 0.001), which became even stronger when AIA was subtracted from TA (r = 0.98; p < 0.001), suggesting that AIA does not represent bone ash; this was also supported by the lack of correlation between AIA and percentage of visible bone, and the negative correlation between AIA and fecal Ca and P (r = −0.34; p = 0.0464). There was no clear correlation between fecal Ca and P and NDIA (Table 2), suggesting that bone ash is not a major constituent of NDIA.

There was no correlation between fecal animal fiber (incl. ash) and the percentage of visible bone, the percentage of visible skin (Table 2), the sum of visible bone and skin, or the sum of fecal Ca and P (Table 2) across diets. Regarding microbial correlations, Fusobacteria had a negative relationship with fecal TA (r = −0.37; p = 0.0333). In contrast, Proteobacteria showed a positively relationship with fecal TA (r = 0.41; p = 0.0158), TA minus AIA (r = 0.36; p = 0.0387), and the sum of fecal Ca and P (r = 0.35; p = 0.0438).

## Discussion

Our study shows the potential impact of raw diet form (i.e., meat on bone or carcass with or without skin/hair) on the gut microbiota and fecal characteristics in zoo carnivores. First, Firmicutes was the most abundant bacterial phylum regardless of the diet type, which is consistent with previous studies on the gut microbiota of felids, such as tigers (*Panthera tigris*) [27,28]; cheetahs (*Acinonyx jubatus*) [12]; snow leopards (*Panthera uncia*) [29]; bobcats (*Lynx rufus*) [30] and domestic cats (*Felis catus*) [13,31]. When fed carcass with skin and hair, the relative abundance of Proteobacteria decreased significantly. The abundance of members of the Proteobacteria is often increased in carnivorous individuals, such as cats, with intestinal inflammation, as they include many clinically important gastrointestinal pathogens [32]. Its impact may vary with diet type, as pet food and raw feeding can influence the microbial composition differently. Despite the presence of *Escherichia-Shigella* as the dominant bacteria, the four lions participating in our study were clinically healthy and showed no signs of gastrointestinal disease. Meanwhile, the abundance of Fusobacteria and Actinobacteria increased after feeding carcass with skin and hair. *Fusobacterium* appears to be associated with inflammatory bowel disease (IBD) and colorectal cancer in humans [33], but not necessarily in carnivores [34]. In healthy carnivore hosts, concentrations of *Fusobacterium* are generally high [35]. Common bacterial communities of the Actinobacteria include the genera *Collinsella* and *Bifidobacterium*. *Bifidobacterium*, a class of intestinal bacteria that can ferment dietary fiber, is often found at high relative abundance in the feces of domestic cats fed a carbohydrate-rich extruded kibble diet [36]. However, no *Bifidobacterium* was detected in the feces of zoo lions fed a strict carnivorous diet in this study, a result consistent with the phylogenetic analysis of the fecal microbiota of zoo cheetahs fed raw food, hence a low carbohydrate diet [12]. *Collinsella* was the most common genus among Actinobacteria in the feces of lions. *Collinsella* plays a role in altering intestinal cholesterol absorption, reducing hepatic glycogen production, and increasing triglyceride synthesis in humans, and its abundance decreased with increasing fiber-rich diets [37]. The composition of animal fiber is different from that of plant fiber, which may affect the gut microbiota through different metabolic pathways. While many associations between specific microbiota and health effects are derived from humans or non-carnivorous species, it is necessary to consider that these relationships may differ in carnivores due to their different dietary characteristics.

One practical limitation of this study is the timing of fecal sample collection. The generally accepted gold standard for fecal microbiome studies is to collect fresh fecal samples within 24 hours and freeze them at −80°C as soon as possible [38,39]. Previous studies have shown that cheetah fecal microbiota exhibits a certain stability during degradation, and although the relative abundance of Fusobacteria and Proteobacteria decreases and increases over the outdoor storage time, respectively, the richness and diversity of the bacterial community stay relatively consistent [40]. In our study, since it was impossible to accurately determine the defecation time of specific individual samples, the dispersion between individuals in the same diet group was analyzed by variance analysis. Variance analysis showed that within-group variability was moderate for dominant taxa (e.g., Firmicutes), whereas some less abundant taxa exhibited higher dispersion (S1 Table). Sequencing depth averaged 30,534 ± 13,748 reads per sample, which may also contribute to sample-to-sample variation. It was found that the groups with higher dispersion were mainly individuals fed the BC and BM02 diets. This may reflect the greater influence of environmental factors, especially in the later period of the experiment (July to August) when the ambient temperature and humidity gradually increased. Exact defecation times could not be determined in the zoo setting, so no statistical test could be performed to assess this effect. Nevertheless, previous studies in free-ranging grizzly bears demonstrated that bacterial DNA in fecal samples remains stable for up to several weeks post-defecation [41], suggesting that moderate delays in sampling are unlikely to have significant biased our microbiota results. It is reasonable to assume that diet was still the main factor driving changes in fecal microbiota. Although the lions were returned to the same beef on meat diet after the carcass feeding, the fecal microbiota composition did not fully revert to the original profile observed at BM01. This indicates that dietary shifts may induce persistent alterations in the gut microbiota, even over relatively short time frames. Similar findings have been reported in dogs and wolves, where the microbiome was shown to require an extended period to reach a new stable state following dietary changes [42].

The main changes in fecal microbiota and characteristics observed in this study occurred after feeding the BC diet. In terms of food composition, the BC diet provided additional types of animal fiber; in particular, the lions ingested skin and hair from the carcass. To more accurately quantify the fiber content in animal carcasses, our group developed the aNDRa method that can specifically measure insoluble fiber, including bone, skin, and hair [19]. This method provides a tool for studying the role of animal fiber in regulating gut health. Due to the large volume of carcasses and leftover food, which are difficult to homogenize, this study only quantified animal fiber in feces, but did not directly measure the intake of animal fiber in the diet. Therefore, we cannot accurately compare the actual intake of animal fiber under different dietary conditions. The total amount of animal fiber in feces was highest on the BC diet, suggesting that the lions consumed more animal fiber on BC.

The fecal bulk basically doubled on BC (Table 1), with feces containing large amounts of undigested skin. This is consistent with other study of carnivores fed various fiber sources [10]. A reasonable hypothesis is that the undigested skin and hair in the feces had a diluting effect on other components. Incompletely digested skin may increase the volume of feces in the form of physical filler, resulting in a decrease in the relative proportion of bone fragments in the feces. Therefore, the reduction in visible bones in the feces of the BC might not necessarily indicate a lower bone intake, but dilution of the relative content of bones in the feces by a large amount of undigested skin. Nevertheless, the total weekly Ca and P excretion suggests a generally lower bone intake on BC, which may be related to differences in the bony parts between the carcass feedings that were not recorded; e.g., a carcass piece containing the thorax might, via ribs, provide more easily chewable bones than a carcass piece of hindquarters.

Bones are rich in minerals, with 60–70% of their dry weight consisting of hydroxyapatite (Ca₁₀(PO₄)₆(OH)₂) hence containing high levels of Ca and P [43]. The changes in total Ca and P after dilution correction support this hypothesis. When looking at individual fecal samples from BC individually, some still had high Ca and P levels even though no visible bones were detected (data not shown). This phenomenon may be related to changes in feeding behavior: the presence of skin may prompt lions to chew carcass tissue more thoroughly, thereby crushing bones into smaller pieces, making them less easily identified by the naked eye in feces, but still reflecting the digestion and excretion of bones through Ca and P determination. Even though the banteng carcass led to the largest mean fecal particle size, it might thus nevertheless have elicited more chewing activity. This hypothesis needs to be tested in future studies.

Diet particle size may affect the gastric emptying rate and gastrointestinal motility pattern. In studies of domestic cats, no significant differences were shown in the apparent digestibility of minced and whole prey [13], suggesting that particle size has a relatively limited effect on the enzymatic digestion of animal foods. However, larger food particles generally take longer to pass through the stomach because they require more complete acid digestion and pyloric peristalsis before entering the small intestine [44]. Adding 10% insoluble fiber (such as sugarcane fiber) to the diet significantly delayed gastric emptying and colon filling time in dogs [45]. Due to the high toughness of the skin, its mechanical processing and digestion take longer, which may increase the physical filling of the stomach and prolong the feeling of fullness. Behavioral observations in parallel to the feeding experiment would be needed to assess whether changes compatible with longer satiety are evident.

By contrast, larger particles in the hindgut might trigger more peristalsis, resulting in faster excretion and less time available for water reabsorption. This is supported by the association of larger fecal particles with lower fecal DM. Larger undigested particles may additionally reduce the direct contact of the gut microbiota with fermentable substrates (such as protein or other organic matter), thus changing the microbial fermentation pattern both due to a reduction in the time available for hindgut fermentation as well as a reduced access of microbes to fermentable substance [46]. In the present study, this could have been a factor for the change in microbe composition between CC and BC towards less Proteobacteria, and more Fusobacteria and Actinobacteria.

Dual fecal consistency – where firm and soft components alternate in fecal excretion of the same individual, either within or between scats – has been observed in several carnivores [47–49]. Structural complexity in the diet can promote the occurrence of this phenomenon, possibly due to variations in retention time and separation mechanisms within the digestive tract. Fecal composition appeared to vary depending on the predominant undigested components, with drier feces typically associated with higher proportions of bone, total ash, and the minerals Ca and P, while feces containing larger amounts of skin tended to retain more moisture. This pattern may reflect differences in packing density, as finely ground bone matrix is often observed in carnivore feces, contributing to the formation of compact, dry, and typically white scats [50]. In contrast, the presence of large, less fragmented pieces of skin may prevent dense fecal packing, leading to a softer consistency. Such differences in fecal characteristics show the impact of animal fiber components on the digestive and mechanical processing in the carnivore gut.

Unexpectedly, there was no significant correlation between animal fiber content and visible bone and skin, nor between animal fiber and fecal Ca and P content. Several factors may explain this. First, visible bone does not represent all undigested bone, and the actual proportion of different animal fiber components in feces remains unknown. Furthermore, while skin and hair contribute to the animal fiber in feces, their mineral content is much lower than that of bones [51,52], which may weaken the expected correlation between animal fiber and Ca and P levels.

The choice of correction method in animal fiber analysis may also influence the result. Initially, we subtracted the ash content in animal fiber for correction. It was assumed that the ash content in animal fiber was mainly derived from bone ash. While it is known that bone ash is soluble in acid [53], well reflected in the negative relationship between fecal AIA and Ca and P contents, we had expected bone ash to be retained after neutral detergent solution. However, NDIA did not show a strong positive correlation with total Ca and P content, indicating that its source is not limited to bone ash, or does not include bone ash systematically. It may also include other indigestible ash sources, such as soil contamination (i.e., AIA) or ash residues in biological matrices. Generally, NDIA was of a higher magnitude than AIA, indicating that it contains, but is not limited to, AIA. Both TA minus NDIA and TA minus AIA showed strong positive correlations with Ca and P contents, indicating that they are more accurate estimates of the contribution of bone ash because bone ash is not represented systematically in either NDIA or AIA. Importantly, ash-corrected animal fiber still showed a significant correlation with NDIA, suggestion that both the non-NDIA residue and the NDIA itself derive from a similar source that remains to be identified. If animal fiber is used as an indicator of the indigestible components of a carnivore diet, including the ash portion (i.e., not correcting for it) may be more appropriate. If animal fiber is used as an indicator of components that cannot be digested by the animal but may be subjected to microbial fermentation in the hindgut, then correcting for NDIA still represents a reasonable approach. How this fraction correlates to functional measures of microbial activity, such as short-chain fatty acids, remains to be investigated. In the present study, ash-corrected animal fiber was not correlated with the frequency of a certain bacterial genera.

When carcasses were fed (CC and BC), feces showed elevated levels of AIA, likely due to increased ingestion of attached soil from large carcasses. Typically, animals can handle a reasonable proportion of soil intake [54,55], and as an indigestible component, soil may contribute to an adequate feces consistency [56]. Additionally, the presence of furred skin in the BC diet may have influenced feeding behavior, potentially leading to prolonged feeding times. A long-term study similarly found that lions fed whole calf carcasses spent more time feeding on average [6]. Although replicating natural hunting and feeding patterns in captivity is challenging, the provision of large carcasses has been observed in some zoos to promote foraging behaviors, reduce stereotyped pacing, and enhance social interactions in carnivores [57,58].

## Conclusion

Our study shows that carcass type influences the fecal microbiome and fecal characteristics of zoo-housed Asiatic lions. Banteng carcass with skin/fur led to the highest excretion of animal fiber and the largest fecal particle size, with animal fiber positively associated with particle size but not with bone intake proxies. Correlation analysis further indicated that bone- and skin-derived components had opposite relationships with fecal DM and ash, while microbial groups such as Fusobacteria and Proteobacteria responded differently to ash parameters. Overall, carcass feeding is not a uniform concept; distinct tissue components such as bone, skin, and hair exert differential effects on gut microbiota and fecal traits.

## Supporting information

S1 Fig Individual relative abundance of bacterial phyla in fecal samples of zoo-housed Asiatic lions fed four different raw diets: beef meat on bone (BM01 and BM02), cattle carcass (CC), and banteng carcass (BC). The top 10 most abundant phyla across all samples are shown; less abundant phyla were grouped under “Other.” Each bar represents one individual sample.
(PDF)

S2 Fig Individual relative abundance of bacterial genera in fecal samples of zoo-housed Asiatic lions fed four different raw diets: beef meat on bone (BM01 and BM02), cattle carcass (CC), and banteng carcass (BC). The top 20 most abundant genera across all samples are shown; less abundant genera were grouped under “Other.” Each bar represents one individual sample.
(PDF)

S3 Fig Changes in relative abundance of fecal microbiota at genus level in feces of zoo-housed Asiatic lions fed beef meat on bone (BM01 and 02), cattle carcass (CC), and banteng carcass (BC). Multiple testing was performed using the Mann Whitney U tests with Benjamini–Hochberg false discovery rate correction (Adjusted *p values* are shown above each comparison).
(PDF)

S1 Table Mean relative abundances (%), standard deviations (SD), and coefficients of variation (CV) of fecal bacterial phyla and genera in zoo-housed Asiatic lions fed four different raw diets (BM01 and BM02: beef meat on bone; CC: cattle carcass; BC: banteng carcass). (DOCX)

S2 Table Ash in fecal matter a collected from zoo-housed Asiatic lions fed four different raw diets (BM01 and BM02: beef meat on bone; CC: cattle carcass; BC: banteng carcass). (DOCX)
