# Supplementary figures and images for: Cuts or carcasses? Diet form affects fecal microbial and animal fiber fractions in a large carnivore, the Asiatic lion
Source: PLoS One. 2025 Oct 22;20(10):e0335173. doi: 10.1371/journal.pone.0335173 (PMC12543182; doi:10.1371/journal.pone.0335173)

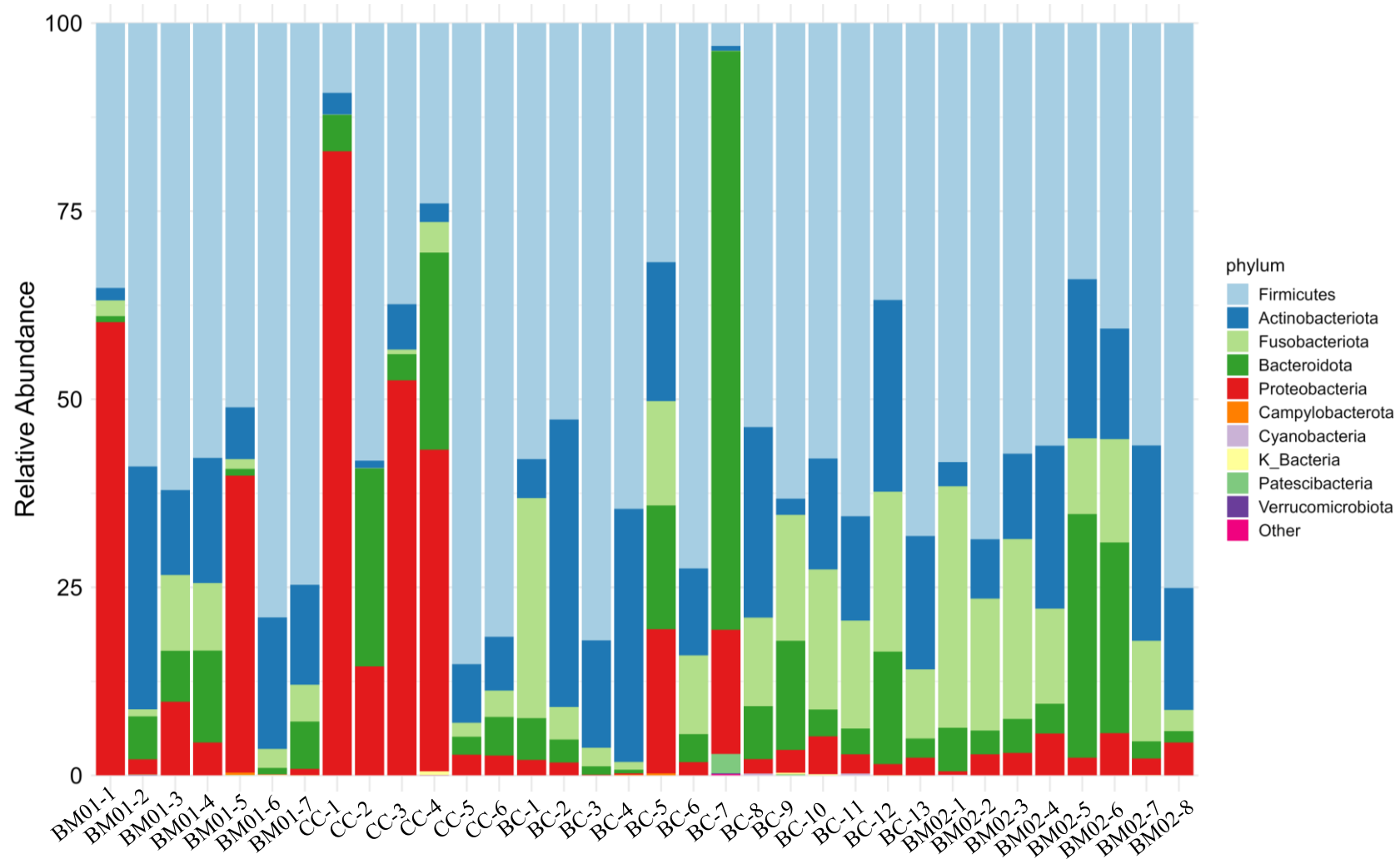

Supplement: S1 Fig — The top 10 most abundant phyla across all samples are shown; less abundant phyla were grouped under “Other.” Each bar represents one individual sample. (PDF) [file pone.0335173.s001.pdf]

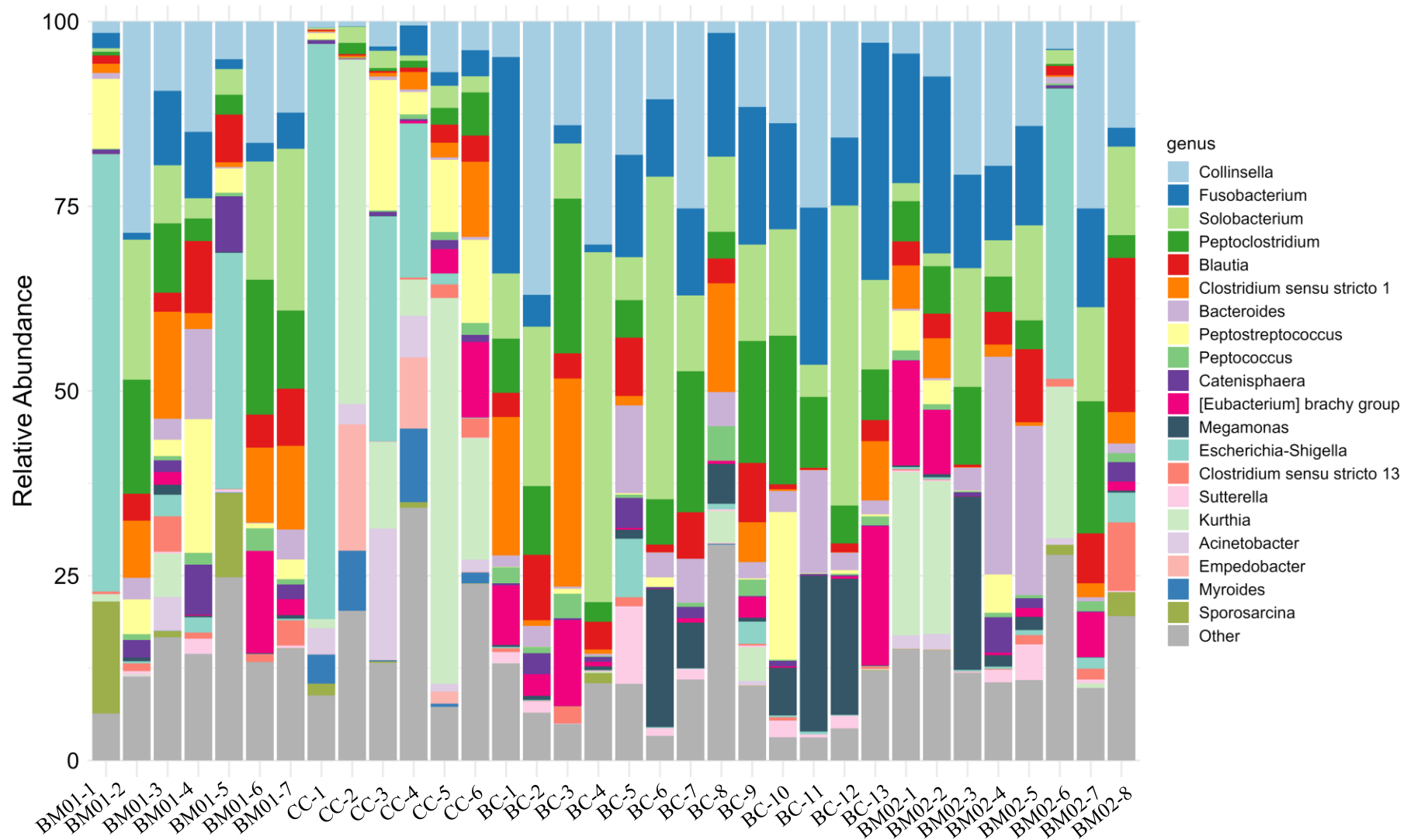

Supplement: S2 Fig — The top 20 most abundant genera across all samples are shown; less abundant genera were grouped under “Other.” Each bar represents one individual sample. (PDF) [file pone.0335173.s002.pdf]

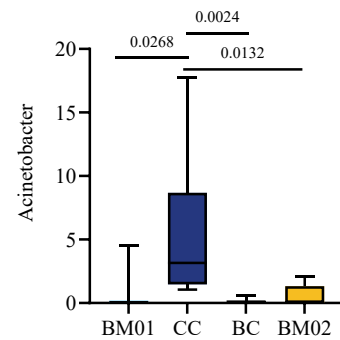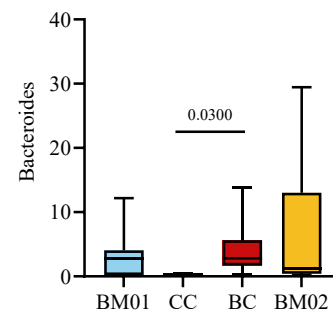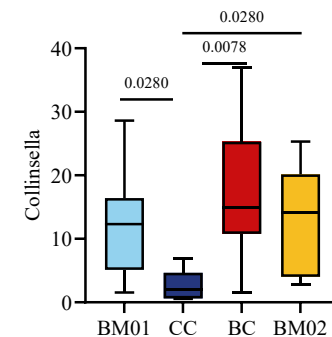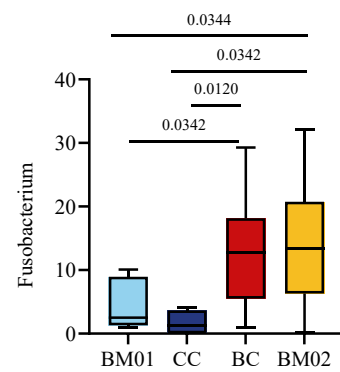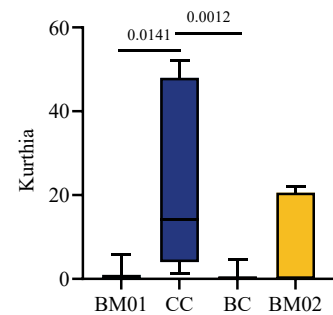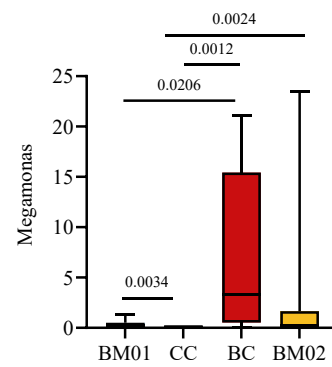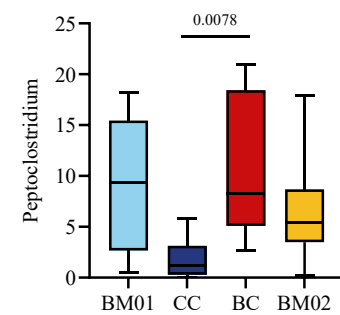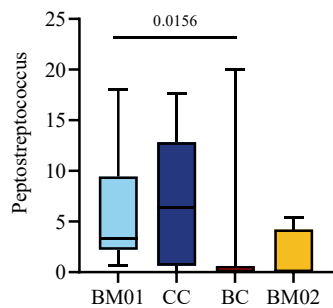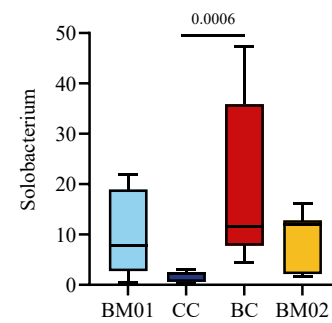

Supplement: S3 Fig — Multiple testing was performed using the Mann Whitney U tests with Benjamini–Hochberg false discovery rate correction (Adjusted p values are shown above each comparison). (PDF) [file pone.0335173.s003.pdf]
