# Supplementary material for: Cuts or carcasses? Diet form affects fecal microbial and animal fiber fractions in a large carnivore, the Asiatic lion
Source: PLoS One. 2025 Oct 22;20(10):e0335173. doi: 10.1371/journal.pone.0335173 (PMC12543182; doi:10.1371/journal.pone.0335173)
Supplement: S1 Table — (DOCX) [file pone.0335173.s004.docx]

**Supplemental Table 1**. Mean relative abundances (%), standard deviations (SD), and coefficients of variation (CV) of fecal bacterial phyla and genera in zoo-housed Asiatic lions fed different raw diets (BM01 and BM02: beef meat on bone; CC: cattle carcass ; BC :banteng carcass).

|  | BM01 | | | CC | | | BC | | | BM02 | | |
| --- | --- | --- | --- | --- | --- | --- | --- | --- | --- | --- | --- | --- |
|  | Mean | SD | CV% | Mean | SD | CV% | Mean | SD | CV% | Mean | SD | CV% |
| **Phylum** |  |  |  |  |  |  |  |  |  |  |  |  |
| Actinobacteriota | 14.2 | 9.7 | 68.2 | 4.6 | 2.8 | 61.7 | 17.0 | 11.4 | 66.8 | 14.1 | 7.9 | 55.9 |
| Bacteroidota | 4.8 | 4.3 | 88.9 | 11.4 | 11.6 | 101.4 | 11.8 | 20.3 | 172.2 | 8.9 | 11.6 | 130.1 |
| Campylobacterota | 0.1 | 0.1 | 220.2 | 0.0 | 0.0 | - | 0.0 | 0.1 | - | 0.0 | 0.0 | - |
| Cyanobacteria | 0.0 | 0.0 | - | 0.0 | 0.0 | - | 0.0 | 0.1 | - | 0.0 | 0.0 | - |
| Firmicutes | 59.8 | 14.6 | 24.4 | 49.3 | 31.0 | 62.9 | 54.6 | 20.6 | 37.7 | 53.7 | 14.0 | 26.1 |
| Fusobacteriota | 4.4 | 3.7 | 85.1 | 1.7 | 1.8 | 104.6 | 11.8 | 8.6 | 72.7 | 14.0 | 9.8 | 70.0 |
| K_Bacteria | 0.0 | 0.0 | - | 0.1 | 0.2 | 240.5 | 0.0 | 0.1 | - | 0.0 | 0.0 | - |
| Patescibacteria | 0.0 | 0.0 | - | 0.0 | 0.0 | - | 0.2 | 0.7 | 337.7 | 0.0 | 0.0 | - |
| Proteobacteria | 16.7 | 23.7 | 141.8 | 33.0 | 32.1 | 97.3 | 4.4 | 6.1 | 137.1 | 9.3 | 18.0 | 194.3 |
| Verrucomicrobiota | 0.0 | 0.0 | - | 0.0 | 0.0 | - | 0.0 | 0.0 | - | 0.0 | 0.0 | - |
| **Genus** |  |  |  |  |  |  |  |  |  |  |  |  |
| Acinetobacter | 0.7 | 1.7 | 234.0 | 5.4 | 6.3 | 116.3 | 0.1 | 0.2 | 304.1 | 0.5 | 0.9 | 160.6 |
| Bacteroides | 3.3 | 4.2 | 127.2 | 0.3 | 0.2 | 63.9 | 4.4 | 4.3 | 99.5 | 6.7 | 11.2 | 165.9 |
| Blautia | 5.1 | 3.0 | 59.5 | 1.2 | 1.4 | 119.0 | 4.0 | 3.1 | 76.6 | 5.9 | 6.3 | 107.2 |
| Catenisphaera | 3.0 | 3.0 | 100.4 | 0.6 | 0.5 | 77.7 | 0.9 | 1.3 | 139.6 | 1.1 | 1.6 | 148.0 |
| Clostridium sensu stricto 1 | 6.8 | 5.5 | 80.8 | 2.6 | 3.8 | 149.9 | 5.8 | 9.5 | 162.1 | 3.1 | 2.9 | 93.6 |
| Clostridium sensu stricto 13 | 1.6 | 1.8 | 107.5 | 0.8 | 1.1 | 143.7 | 0.4 | 0.7 | 178.3 | 1.5 | 2.9 | 192.7 |
| Collinsella | 12.6 | 8.8 | 69.7 | 2.7 | 2.5 | 93.8 | 17.3 | 10.4 | 60.1 | 12.5 | 8.3 | 66.3 |
| Empedobacter | 0.0 | 0.0 | - | 4.8 | 7.1 | 149.6 | 0.0 | 0.0 | - | 0.0 | 0.0 | - |
| Escherichia-Shigella | 13.8 | 23.1 | 167.2 | 21.8 | 30.2 | 138.7 | 1.1 | 2.3 | 212.9 | 5.2 | 12.9 | 247.6 |
| Fusobacterium | 4.4 | 3.7 | 85.1 | 1.7 | 1.8 | 104.6 | 12.8 | 8.2 | 63.9 | 14.0 | 9.9 | 70.7 |
| Kurthia | 1.0 | 2.2 | 218.0 | 22.2 | 21.8 | 98.2 | 0.8 | 1.7 | 226.6 | 7.1 | 10.5 | 147.7 |
| Megamonas | 0.4 | 0.5 | 117.6 | 0.0 | 0.0 | - | 6.6 | 8.1 | 122.2 | 3.1 | 7.7 | 247.6 |
| Myroides | 0.0 | 0.0 | - | 4.0 | 4.2 | 103.7 | 0.0 | 0.0 | - | 0.0 | 0.0 | - |
| Peptoclostridium | 8.6 | 6.8 | 79.2 | 1.8 | 2.1 | 116.8 | 10.5 | 6.8 | 65.1 | 6.6 | 5.1 | 77.4 |

- For taxa with group mean = 0%, CV% is not reported due to instability at near-zero means.
