# Supplementary material for: Cuts or carcasses? Diet form affects fecal microbial and animal fiber fractions in a large carnivore, the Asiatic lion
Source: PLoS One. 2025 Oct 22;20(10):e0335173. doi: 10.1371/journal.pone.0335173 (PMC12543182; doi:10.1371/journal.pone.0335173)
Supplement: S2 Table — (DOCX) [file pone.0335173.s005.docx]

|  | BM01 | CC | BC | BM02 | *P* ^*^ |
| --- | --- | --- | --- | --- | --- |
| Total ash ^b^ (% DM) | 47.1^b^ ± 5.9 | 48.4^b^ ± 5.6 | 24.0^a^ ± 13.0 | 44.6^b^ ± 6.7 | <0.0001 |
| NDIA ^c^ (% animal fiber) | 66.2^ab^ ± 11.8 | 71.5^b^ ± 7.1 | 57.2^a^ ± 12.2 | 68.1^ab^ ± 6.6 | 0.0277 |
| NDIA ^d^ (% DM) | 19.5 ± 6.8 | 25.6 ± 5.1 | 16.5 ± 7.2 | 16.0 ± 8.0 | 0.0578 |
| AIA ^e^ (% total ash) | 14.9^a^ ± 8.0 | 32.2^b^ ± 17.5 | 53.2^c^ ± 12.1 | 19.1^ab^ ± 9.9 | <0.0001 |
| AIA ^f^ (% DM) | 6.7 ± 3.4 | 14.9 ± 7.3 | 12.3 ± 7.1 | 8.4 ± 4.5 | 0.0652 |

**Supplemental Table 2.** Ash in fecal matter ^a^ collected from zoo-housed Asiatic lions fed different raw diets (BM01 and BM02: beef meat on bone; CC: cattle carcass ; BC :banteng carcass).

NDIA, neutral detergent insoluble ash; AIA, acid insoluble ash; DM, dry matter; SD, standard deviation.

^a^ Mean ± SD

^b^ Total ash (% DM of feces)= (Weight of crucibles with ash after ashing - Weight of crucibles before use) / (Weight of feces sample before ashing) x 100

^c^ NDIA (% DM of animal fiber) = (Weight of crucibles with NDIA after ashing - Weight of crucibles before use) / (Weight of neutral detergent residues after amylase treatment in animal fiber) x 100

^d^ NDIA (% DM of feces) = [NDIA (% DM of animal fiber) × animal fiber content (% DM of feces)]/100

^e^ AIA (% DM of total ash) = (Weight of the sample tube with AIA after centrifuging and drying – Weight of the sample tube before its use)/(Weight of ash used for mixing with 2 N HCl) x 100

^f^ AIA (% DM of feces) = [AIA (% DM of total ash) × total ash (% DM of feces)]/100

^*^Data were analyzed by one-way ANOVA at a significance level of p < 0.05. Values in the same row with the same superscript do not differ significantly. Superscripts represent post hoc Tukey pairwise comparisons among the four diet groups.
